# Distinct clinical and biological characteristics of acute myeloid leukemia with higher expression of long noncoding RNA *KIAA0125*

**DOI:** 10.1007/s00277-020-04358-y

**Published:** 2020-11-23

**Authors:** Yu-Hung Wang, Chien-Chin Lin, Chia-Lang Hsu, Sheng-Yu Hung, Chi-Yuan Yao, Sze-Hwei Lee, Cheng-Hong Tsai, Hsin-An Hou, Wen-Chien Chou, Hwei-Fang Tien

**Affiliations:** 1 Graduate Institute of Clinical Medicine, College of Medicine, National Taiwan University, Taipei, Taiwan; 2 Division of Hematology, Department of Internal Medicine, National Taiwan University Hospital, No. 7, Chung-Shan S. Rd, Taipei City, 10002 Taiwan; 3 Department of Laboratory Medicine, National Taiwan University Hospital, No. 7, Chung-Shan S. Rd, Taipei City, 10002 Taiwan; 4 Department of Medical Research, National Taiwan University Hospital, Taipei, Taiwan; 5 Division of Hematology, Department of Internal Medicine, National Taiwan University Hospital Yunlin Branch, Yunlin, Taiwan; 6 Tai-Cheng Stem Cell Therapy Center, National Taiwan University, Taipei, Taiwan

**Keywords:** Long non-coding RNA, *KIAA0125*, Acute myeloid leukemia, Chemoresistance, Leukemic stem cell signatures

## Abstract

Expression of long non-coding RNA *KIAA0125* has been incorporated in various gene expression signatures for prognostic prediction in acute myeloid leukemia (AML) patients, yet its functions and clinical significance remain unclear. This study aimed to investigate the clinical and biological characteristics of AML bearing different levels of *KIAA0125*. We profiled *KIAA0125* expression levels in bone marrow cells from 347 de novo AML patients and found higher *KIAA0125* expression was closely associated with *RUNX1* mutation, but inversely correlated with t(8;21) and t(15;17) karyotypes. Among the 227 patients who received standard chemotherapy, those with higher *KIAA0125* expression had a lower complete remission rate, shorter overall survival (OS) and disease-free survival (DFS) than those with lower expression. The prognostic significance was validated in both TCGA and GSE12417 cohorts. Subgroup analyses showed that higher *KIAA0125* expression also predicted shorter DFS and OS in patients with normal karyotype or non-M3 AML. In multivariable analysis, higher *KIAA0125* expression remained an adverse risk factor independent of age, WBC counts, karyotypes, and mutation patterns. Bioinformatics analyses revealed that higher *KIAA0125* expression was associated with hematopoietic and leukemic stem cell signatures and ATP-binding cassette transporters, two predisposing factors for chemoresistance.

**Supplementary Information:**

The online version contains supplementary material available at 10.1007/s00277-020-04358-y.

## Introduction

Long non-coding RNAs (lncRNAs) are non-protein coding RNAs that are longer than 200 nucleotides. Comparing to other classes of ncRNAs, lncRNAs exhibit a wide range of structures and functions [1]. Recently, lncRNAs have emerged as important regulators for gene expression via remodeling nuclear architecture, modulating mRNA stability and translation, and post-translational modifications [1–4]. Besides, some lncRNAs are dysregulated and harbor prognostic relevance in several types of cancers [5–8]. However, the roles of lncRNAs in tumorigenesis are still largely unknown.

In recent years, research on lncRNAs has increased drastically, and the results are robust. Although the functions of lncRNAs have not been elusive, recent studies suggested the expressions of lncRNAs could be used as prognostic factors, predictors of response, and potential therapeutic targets in acute leukemia [9–18]. Moreover, several gene expression-based prognostic scores have been developed for better risk stratification of acute myeloid leukemia (AML) patients [19–24]. Among those high-risk genes, lncRNA gene *KIAA0125* (also named as *FAM30A*), a hematopoietic stem cell gene localized on chromosome 14, is unique because it is the only non-coding gene and is expressed in humans but not in mice (From the UniProt database, https://www.uniprot.org/uniprot/Q9NZY2). Additionally, *KIAA0125* expression was integrated into a recently proposed 17-gene stemness score, which could predict outcomes in AML patients [19].

This study aimed to investigate the association of *KIAA0125* expression with clinical and biological characteristics in AML patients. We first profiled the expression levels of *KIAA0125* in bone marrow (BM) cells from AML patients and normal controls and demonstrated that AML patients had higher *KIAA0125* expression than normal controls. Higher expression of *KIAA0125* was associated with distinct clinical and biological characteristics and served as an independent poor prognostic biomarker for AML patients in ours and two other publicly annotated cohorts. Further bioinformatics analyses showed that higher expression of *KIAA0125* in AML was closely associated with hematopoietic stem cell (HSC) and leukemic stem cell (LSC) signatures and several important ATP-binding cassette transporters (ABC transporters); these factors are regarded responsible for chemoresistance in AML. Further functional studies are needed to unravel its underlying mechanism and pathogenetic role in AML.

## Materials and methods

### Patients

We recruited 347 adult patients with de novo AML diagnosed in the National Taiwan University Hospital (NTUH) from 1996 to 2011 who had enough cryopreserved BM cells for tests. The diagnoses were based on the French–American–British (FAB) and the 2016 World Health Organization classifications [25, 26]. Among them, 227 patients received standard chemotherapy. Non M3 (acute promyelocytic leukemia, APL) patients received idarubicin 12 mg/m^2^ per day days 1–3 and cytarabine 100 mg/m^2^ per day days 1–7, and then consolidation chemotherapy with 2–4 courses of high-dose cytarabine 2000 mg/m^2^ q12h for total 8 doses, with or without an anthracycline (Idarubicin or Mitoxantrone), after achieving complete remission (CR) as described previously [27]. APL patients received concurrent all-trans retinoic acid and chemotherapy. The remaining 120 patients received supportive care and/or reduced-intensity anti-leukemia therapy due to underlying comorbidities or based on the decision of the physicians or patients. BM samples from 30 healthy donors of hematopoietic stem cell transplantation (HSCT) were collected as normal controls. This study was approved by the Research Ethics Committee of NTUH with informed consent obtained from all participants.

### Microarray and genetic alteration analysis

We profiled the global gene expression of BM mononuclear cells from 347 AML patients and 30 healthy transplant donors by Affymetrix GeneChip Human Transcriptome Array 2.0 as described previously [21, 28, 29]. The raw and normalized microarray data reported in this article have been deposited in the Gene Expression Omnibus database (accession number GSE68469 and GSE71014) [21, 28, 29]. For external validation, we analyzed two publicly annotated datasets, the microarray dataset of GSE12417-GPL96 cohort, which includes the gene expression profile of 163 patients with cytogenetically normal AML, and the RNAseq dataset of the TCGA cohort (*n* = 186) [20, 30]. Cytogenetic analyses were performed and interpreted as described previously [31]. We also analyzed the mutation statuses of 17 myeloid-relevant genes, including *ASXL1, IDH1, IDH2, TET2, DNMT3A, FLT3-*ITD, *FLT3-*TKD, *KIT*, *NRAS*, *KRAS*, *RUNX1*, *MLL/*PTD, *CEBPA*, *NPM1*, *PTPN11*, *TP53*, and *WT1* by Sanger sequencing as previously described [27, 28, 31–34].

### Analysis of gene expression in next-generation sequencing datasets

We analyzed gene expression data of 141 AML samples profiled with Illumina Genome Analyzer RNA Sequencing in the TCGA database [30] to investigate the absolute gene expression levels.

### Gene set enrichment analysis

The preranked Gene Set Enrichment Analysis (GSEA) implemented by R package clusterProfiler was performed using the stem cell-related gene sets from the MSigDB databases. The genes were ranked based on the Spearman’s correlation coefficient between the given gene and *KIAA0125*.

### Statistical analysis

We used the Mann-Whitney U test and ANOVA test, where appropriate, to compare continuous variables and medians/means of distributions. The Fisher exact test or the χ2 test was performed to examine the difference in discrete variables, including gender, cytogenetic changes, and genetic alterations between patients with lower and higher *KIAA0125* expression. Overall survival (OS) was the duration from the date of initial diagnosis to the time of last follow-up or death from any cause, whichever occurred first. Disease-free survival (DFS) was the duration from the date of attaining a leukemia-free state until the date of AML relapse or death from any cause, whichever occurred first. The survival prediction power of *KIAA0125* expression was evaluated by both the log-rank test and the univariate Cox proportional hazards model. We plotted the survival curves with Kaplan-Meier analysis and calculated the statistical significance with the log-rank test. To find the optimal cutoff for separating patient groups, we used maximally selected rank statistics implemented in the maxstat R package. The Cox proportional hazards model was used in multivariable regression analysis. *P* values < 0.05 were considered statistically significant. All statistical analyses were performed with BRB-ArrayTools (version 4.5.1; Biometric Research Branch, National Cancer Institute, Rockville, MD), and IBM SPSS Statistics 23 for Windows.

## Results

The median age of the 347 AML patients was 57 years. Among the 331 patients who had cytogenetic data at diagnosis, 165 (49.8%) had clonal chromosomal abnormalities. Sixty patients (18.1%) had favorable cytogenetics; 223 (67.2%), intermediate-risk cytogenetics; and 14.8% unfavorable cytogenetics (Supplement Table 1) based on the refined British Medical Research Council (MRC) classification [35]. The clinical and laboratory characteristics of these patients at diagnosis are summarized in Table 1.

**Table 1:** Comparison of clinical and laboratory features between AML patients with lower and higher BM *KIAA0125* expression

Clinical characters	Total (*N* = 347)	High *KIAA0125* (*n* = 174)	Low *KIAA0125* (*n* = 173)	*P* value
**Sex**				0.174
**Male**	196	92	104	
**Female**	151	82	69	
Age*		57 (15–91)	58 (18–90)	0.830
Laboratory data*				
**WBC, X 10**^**9**^ **/L**	21.9 (0.38–423)	21.4 (0.38–417.5)	22.38 (0.65–423.0)	0.872
**Hb, g/dL**	8.1 (3.3–16.2)	8.1 (3.3–13.2)	8.1 (3.7–16.2)	0.959
**Platelet, X 10**^**9**^ **/L**	45 (2–655)	54 (6–455)	41 (2–655)	0.060
**Blast, X 10**^**9**^ **/L**	9.1 (0–369.1)	12.3 (0–345.9)	5.7 (0–369.1)	0.021
**LDH (U/L)**	917 (202–13,130)	892.5 (242–7734)	925 (202–13,130)	0.787
**Risk groups**				
**t(8;21)**	24	0 (0)	24 (14.3)	< 0.001
**t(15;17)**	27	3 (1.8)	24 (14.3)	< 0.001
**inv(16)**	9	6 (3.7)	3 (1.8)	0.332
***CEBPA***^**double**^	27	13 (48.1)	14 (51.9)	0.829
***NPM1*+/*FLT3*-ITD-**	57	32 (18.4)	25 (14.5)	0.385
***NPM1*-/*FLT3*-ITD+**	19	3 (1.7)	16 (9.2)	0.002
***RUNX1***	50	32 (64)	18 (36)	0.034
***ASXL1***	52	26 (50)	26 (50)	0.982
**Unfavorable karyotypes**†‡	49	30 (18.3)	19 (11.3)	0.089
**Induction response, n (%)**	227	116	111	
**CR**	165 (72.7)	71 (61.2)	94 (84.7)	< 0.001
**PR**	5 (2.2)	4 (3.4)	1 (0.9)	0.191
**Refractory**	42 (18.5)	33 (28.4)	9 (8.1)	< 0.001
**Induction death**	15 (6.6)	8 (6.9)	7 (6.3)	0.858
**Relapse (%)**	72 (31.7)	42 (36.2)	30 (27.0)	0.137

Abbreviations: *CR* complete remission, *Hb* hemoglobin, *HSCT* allogeneic hematopoietic stem cell transplantation, *LDH* lactate dehydrogenase, *PR* partial remission

*Median (range)

†Cytogenetic data at diagnosis were available in 332 patients, including 168 with lower *KIAA0125* expression and 164 with higher *KIAA0125* expression

‡Based on the refined Medical research Council (MRC) classification

### Comparison of clinical characteristics and genetic alterations between patients with higher and lower KIAA0125 expression

**Fig. 1:**
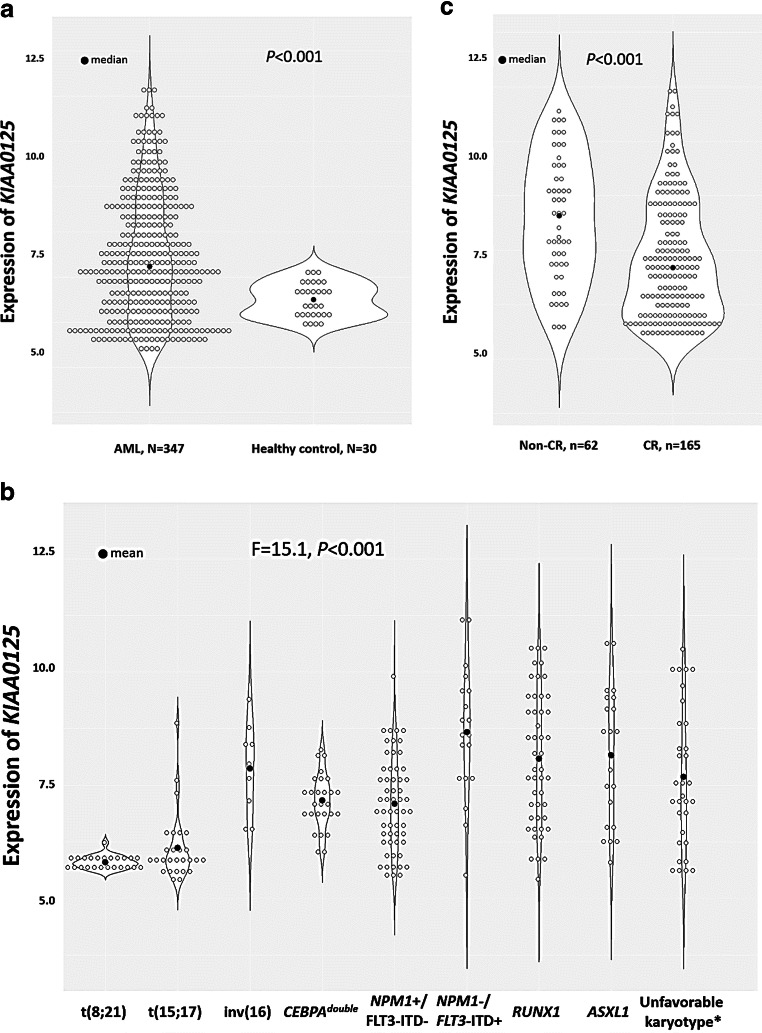
**Dot plots depicting expression levels of** ***KIAA0125*** **in healthy controls and various AML subgroups. a** Patients with AML had significantly higher expression of *KIAA0125* than healthy controls; **b** patients with karyotypes of t(8;21) or t(15;17) had significantly lower expression of *KIAA0125* than any other subgroups while patients with *NPM1*-/*FLT3*-ITD+, *RUNX1*, *ASXL1*, or unfavorable karyotypes had highest expression among all subgroups; and **c** patients who achieved CR after induction chemotherapy had lower expression of BM *KIAA0125* at diagnosis than those who did not. *Based on the refined Medical research Council (MRC) classification

The distribution of *KIAA0125* expression of 347 AML patients is shown with dot plots in Supplement Fig. 1. We first compared the BM *KIAA0125* expression between the 30 healthy controls and 347 AML patients. The expression of *KIAA0125* was significantly higher in AML samples than healthy controls (*p* < 0.001, Fig. 1a). Then, the 347 AML patients were divided into two groups by the median value of the *KIAA0125* expression. The comparison of clinical and laboratory features between the two groups is shown in Table 1. The higher-*KIAA0125* group had higher circulating blasts at diagnosis (*p* = 0.021) and higher incidence of *FLT3*-ITD in the absence of *NPM1* mutation (*NPM1*-/*FLT3*-ITD+) (*p* = 0.002) and *RUNX1* mutation (*p* = 0.034), but lower incidence of t(8;21) and t(15;17) (both *p* < 0.001), compared with the lower-*KIAA0125* group (Table 1). From another perspective, patients with t(8;21) or t(15;17) had lower *KIAA0125* expression, whereas those with *RUNX1* mutation, *ASXL1* mutation, *NPM1*-/*FLT3*-ITD+, or unfavorable karyotypes had higher expression of *KIAA0125* (F = 15.124, *p* < 0.001, Fig. 1b, Supplement Table 1 and Supplement Table 2). Furthermore, the association of higher-*KIAA0125* with lower frequencies of t(8;21) and t(15;17) was observed in both the NTUH cohort (both *p* < 0.001, Supplement Table 3) and TCGA cohort (*p* = 0.006 and *p* < 0.001, respectively, Supplement Table 3). The higher-*KIAA0125* patients more frequently had *FLT3*-ITD (*p* = 0.048) and mutations in *DNMT3A* (*p* = 0.015) and *RUNX1* (*p* = 0.034) (Supplement Table 4). Compatible with this finding, patients with *DNMT3A* or *RUNX1* mutation had higher *KIAA0125* expression than those without the mutation (*p* = 0.019 and 0.045, respectively, Supplement Fig. 2). Similarly, there was close association between higher *KIAA0125* expression and *DNMT3A* (*p* = 0.001) and *RUNX1* mutations (*p* = 0.017) in the TCGA cohort (Supplement Table 5). Among the 227 patients who received standard chemotherapy, 165 (72.7%) patients attained a complete remission (CR), while 42 (18.5%) patients had primary refractory diseases. Notably, the patients with higher *KIAA0125* expression had a lower CR rate (61.2% vs. 84.7%, *p* < 0.001) than those with lower expression. In accordance with this finding, the patients who achieved CR after induction chemotherapy had lower expression of BM *KIAA0125* at diagnosis than those who did not (*p* < 0.001, Fig. 1c).

### The impacts of the KIAA0125 expression on OS and DFS

**Fig. 2:**
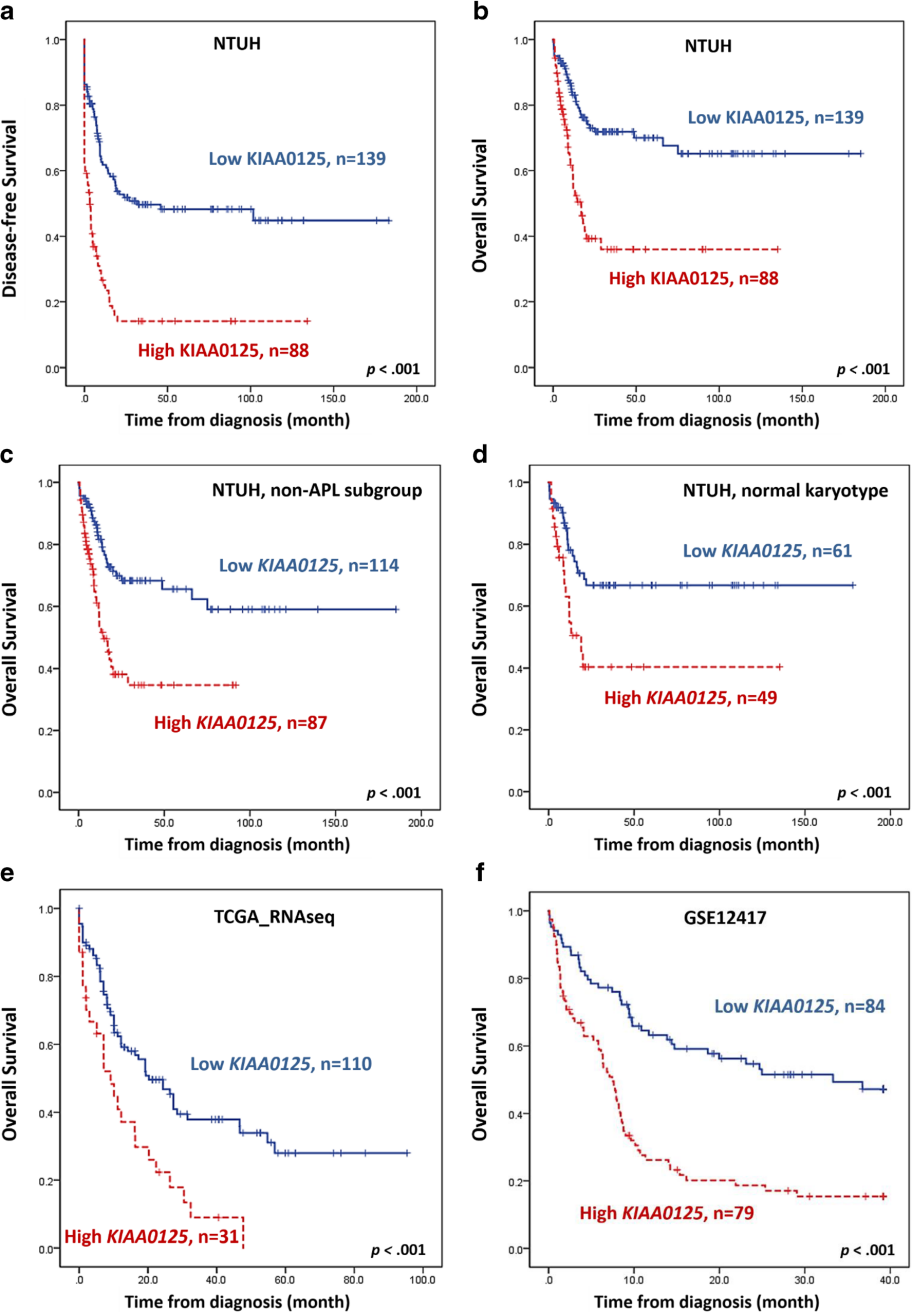
**Kaplan-Meier survival curves stratified by expression of** ***KIAA0125*.** DFS **a** and OS **b** of the 227 AML patients receiving standard chemotherapy in the NTUH cohort; OS of 201 non-APL patients **c** and 110 cytogenetically normal AML patients **d** who received standard treatment in the NTUH cohort; and OS of 141 patients in the TCGA cohort **e** and GSE12417-GPL96 cohort **f**. Patients with higher *KIAA0125* expression had worse clinical outcomes than those with lower expression

Next, we divided patients into two groups with high and low *KIAA0125* expression with cut points determined by the maximally selected rank statistics (7.72 in the NTUH cohort, 8.56 in the TCGA cohort, and 9.71 in GSE12417 cohort, respectively, Supplement Fig. 3). As expected, patients with higher *KIAA0125* expression had an inferior DFS and OS than those with lower expression, no matter whether the survival was censored on the day of hematopoietic stem cell transplantation (HSCT) (median, 3.2 months vs. 31.7 months, *p* < 0.001; and 17 months vs. not reached (NR), *p* < 0.001, respectively; Fig. 2a and b) or not (*p* < 0.001 and *p* < 0.001, respectively; Supplement Fig. 4a and 4b). Subgroup analyses showed that the prognostic significance of *KIAA0125* expression for DFS and OS remained valid in both non-APL and normal karyotype patients (Figs. 2c and d).

In multivariable analysis, we included clinically relevant parameters and variables with a *p value* < 0.05 in univariate Cox regression analysis (Supplement Table 4) as covariates, including age, white blood cell counts at diagnosis, karyotypes, mutation statuses of *NPM1*/*FLT3*-ITD, *CEBPA*^double mutations^, *RUNX1*, *MLL-PTD*, and *TP53*, and *KIAA0125* expression. Higher *KIAA0125* expression, either divided by the selected cut-point (Table 2) or calculated as continuous values (Supplement Table 5), was an independent adverse prognostic factor for DFS (*p* < 0.001 and *p* < 0.001, respectively) and OS (*p* = 0.003 and *p* = 0.001, respectively). To verify the prognostication power of the *KIAA0125* expression, we analyzed the expression of *KIAA0125* and its prognostic significance in the TCGA cohort and the GSE12417-GPL96 cohort. Consistent with the findings in the NTUH cohort, patients with higher *KIAA0125* expressions had a significantly shorter OS (9.2 months vs. 20.3 months, *p < 0.001*, and 7.4 months vs. 33.3 months, *p* < 0.001, respectively, Figs. 2e and f) than those with lower *KIAA0125* expression in the two external validation cohorts.

**Table 2:** Multivariable analysis for DFS and OS in 227 AML patients who received standard intensive chemotherapy

	DFS				OS			
	95% CI				95% CI			
Variable	HR	Lower	Upper	*P*	HR	Lower	Upper	*P*
Age*	1.007	0.995	1.019	0.253	1.030	1.014	1.047	< 0.001
WBC*	1.004	1.002	1.007	0.001	1.005	1.001	1.008	0.012
Karyotype†	1.610	1.201	2.160	0.001	1.706	1.158	2.513	0.007
*NPM1/FLT3-*ITD‡	0.601	0.332	1.089	0.093	0.895	0.443	1.808	0.757
*CEBPA*^double^	0.598	0.286	1.252	0.173	0.451	0.137	1.488	0.191
*RUNX1*	1.532	0.875	2.683	0.136	1.432	0.726	2.821	0.300
*MLL*-PTD	2.706	1.263	5.799	0.010	2.882	1.077	7.710	0.035
*TP53*	1.918	0.697	5.283	0.207	3.030	0.956	9.608	0.060
Higher *KIAA0125* expression§	2.300	1.569	3.371	<0.001	2.188	1.317	3.636	0.003

*p* values < .05 are considered statistically significant

Abbreviations: HR, hazard ratios; CI, confidence interval

*As continuous variable

†Unfavorable cytogenetics versus others. The classification of favorable, intermediate and unfavorable cytogenetics is based on the refined Medical Research Council (MRC) classification [27]. Favorable: t(15;17)(q22;q21), t(8;21)(q22;q22), and inv.(16)(p13q22)/t(16;16)(p13;q22); unfavorable: abn(3q) (excluding t(3;5)(q25;q34)), inv.(3)(q21q26)/t(3;3)(q21;q26), add(5q)/del(5q), −5, −7, add(7q)/del(7q), t(6;11)(q27;q23), t(10;11)(p1113;q23), other t(11q23) (excluding t(9;11)(p21 ~ 22;q23) and t(11;19)(q23;p13)), t(9;22)(q34;q11), −17, and abn(17p); and intermediate: entities not classified as favorable or adverse. Seven patients without chromosome data were not included in the analysis

‡*NPM1*+/*FLT3*-ITD- versus other subtypes

§High vs. low expression of *KIAA0125*

### Biological impacts of KIAA0125 in AML

**Fig. 3:**
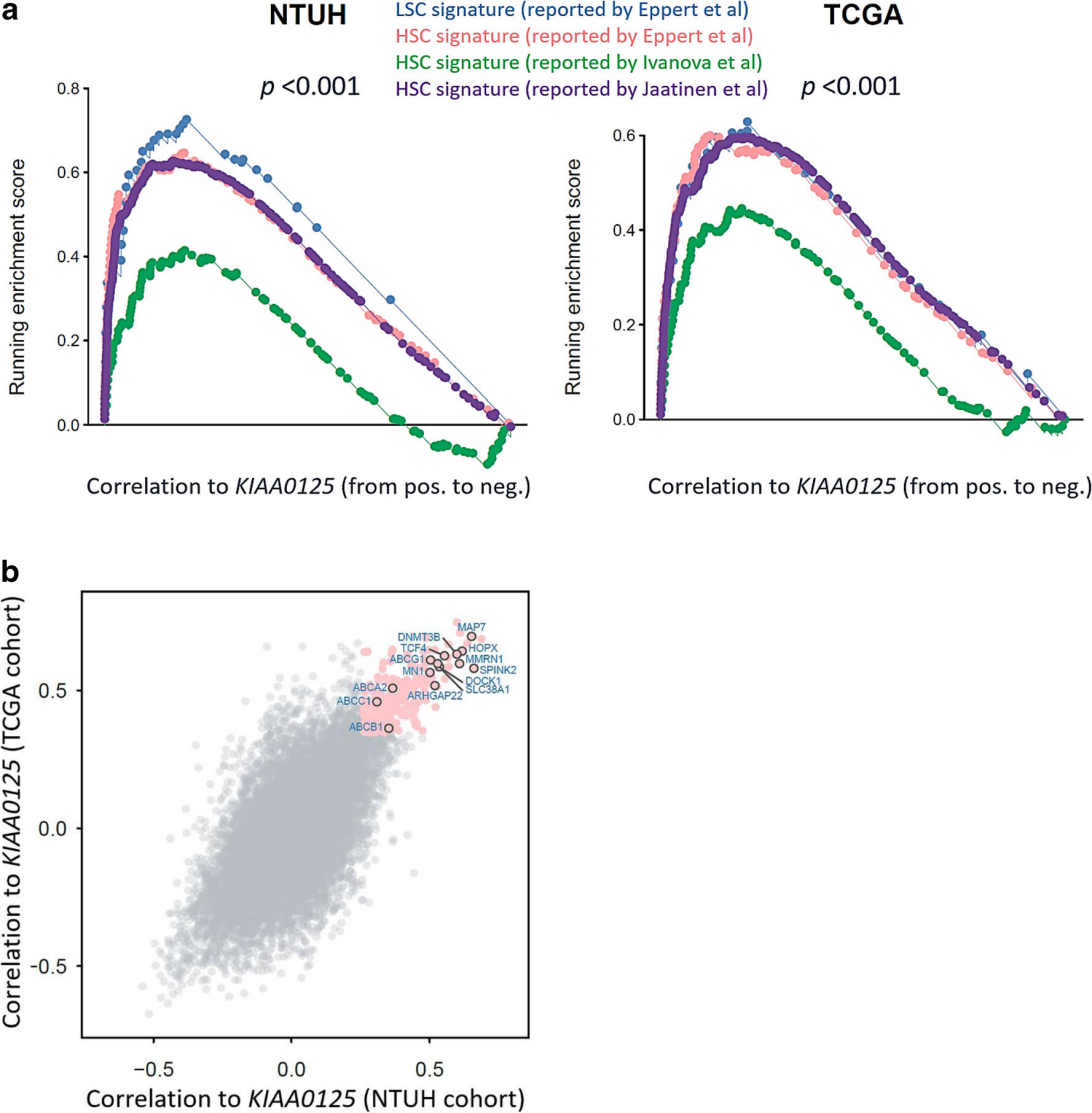
**GSEA enrichment plots of HSC and LSC signatures and scatter plot of genes positively associated with higher** ***KIAA0125*** **expression. a** GSEA enrichment plots show positive association of higher *KIAA0125* expression with HSC and LSC signatures curated from several published reports in both the NTUH and TCGA cohorts; **b** the scatter plot reveals the genes strongly correlated to *KIAA0125* expression in both the NTUH and TCGA cohorts (pink). The correlation measurement is based on the Spearman’s correlation coefficient between the given gene and *KIAA0125*. The strongly correlated genes are defined as their correlation values at top 5% of all genes in both cohorts

To gain biological insights into the underlying mechanism of unfavorable prognosis related to *KIAA0125* overexpression, we investigated the genes whose expression is strongly correlated with that of *KIAA0125*. Since *KIAA0125* was reported as an LSC marker [19], we curated several published HSC and LSC signatures from different studies [36–38]. GSEA showed HSC and LSC signatures were all significantly enriched in the patients with higher *KIAA0125* expression in both the NTUH and TCGA cohorts (both *p* < 0.001, Fig. 3a). We next checked the leading-edge genes whose expression levels were most positively correlated to *KIAA0125* expression in both NTUH and TCGA cohorts. Among them, *SPINK2*, *MAP7*, *HOPX*, *MMRN1*, *DNMT3B*, *TCF4*, *SLC38A1*, *DOCK1*, *ARHGAP22*, *MN1*, and 4 genes in the ATP-binding cassette (ABC) superfamily (*ABCG1*, *ABCA2*, *ABCB1*, and *ABCC1*) have been reported to be associated with poor prognosis or chemoresistance in AML (Fig. 3b and Table 3) [19, 39–58].

**Table 3:** Summary of the biological functions of the *KIAA0125*-associated genes that have been reported to be associated with prognosis or drug resistance in AML patients and their correlation values with *KIAA0125* in ours and the TCGA cohorts

Genes	Correlation coefficient (*p* value)				Association with leukemia
	NTUH		TCGA		
***SPINK2***	0.661	(3.4E-45)	0.5798	(6.2E-15)	Serine Peptidase Inhibitor; upregulation is associated with poor outcomes in adult patients with AML [30]; integrated into a 6-gene LSC score to identifies high risk pediatric AML [31]
***MAP7***	0.653	1.0E-43	0.696	(<E-45)	Microtubule-associated proteins, overexpressed in cytogenetically normal AML patients with dismal outcomes [32]
***HOPX***	0.619	(2.6E-38)	0.643	(<E-45)	The smallest homeodomain protein; higher expression predicts poor prognosis in de novo AML [33]
***MMRN1***	0.609	(9.7E-37)	0.597	(<E-45)	A member of the elastin microfibrillar interface protein; an adverse marker in both pediatric and adult AML [34]
***DNMT3B***	0.599	(1.7E-35)	0.631	(<E-45)	DNA methyltransferases; an important LSC marker [35–37]
***TCF4***	0.556	(1.1E-29)	0.626	(<E-45)	A transcription factor; predict outcome in *RUNX1* mutated and translocated AML [38, 39]
***SLC38A1***	0.536	(2.3E-27)	0.585	(<E-45)	A glutamine amino acid transporter, overexpressed in AML patients with adverse clinical outcomes [40]
***DOCK1***	0.530	(1.1E-26)	0.597	(5.9E-16)	A novel class of guanine nucleotide exchange factors; high expression confers poor prognosis in AML [41]
***ARHGAP22***	0.519	(1.5E-25)	0.518	(<E-45)	Rho GTPase activating protein, incorporated in the 17-gene LSC score which predicts treatment response in AML [9]
***MN1***	0.502	(1.1E-23)	0.565	(<E-45)	A transcriptional coactivator, overexpression could induce AML in mice and predict ATRA resistance in human AML patients [42, 43]
***ABCG1***	0.504	(6.7E-24)	0.610	(<E-45)	Belongs to ATP-binding cassette (ABC) superfamily; responsible for important chemoresistance mechanism in AML [44–49]
***ABCA2***	0.367	(1.5E-12)	0.507	(2.3E-11)	Belongs to ATP-binding cassette (ABC) superfamily; a strong prognostic biomarker for multidrug resistance in pediatric acute lymphoblastic leukemia [44–49]
***ABCB1***	0.353	(1.2E-11)	0.364	(5.2E-6)	Belongs to ATP-binding cassette (ABC) superfamily; responsible for important chemoresistance mechanism in AML [44–49]
***ABCC1***	0.310	(3.2E-9)	0.458	(5.2E-9)	Belongs to ATP-binding cassette (ABC) superfamily; responsible for important chemoresistance mechanism in AML [44–49]

## Discussion

AML cells have abnormal genetic background, either mutations or aberrant expression of specific genes. In recent years, several gene expression scores have been proposed for prognostic prediction of AML patients. We previously developed a 11-gene mRNA expression signature, including *AIF1L*, *CXCR7*, *DNTT*, *GPR56*, *H1F0*, *IFITM3*, *KIAA0125*, *MX1*, *STAB1*, *TM4SF1*, and *TNS3*, for prognostication in AML patients [21]. Another group built a six-gene leukemia stem cell (LSC) score with the incorporation of *DNMT3B*, *GPR56*, *CD34*, *SOCS2*, *SPINK2*, and *KIAA0125* expressions for pediatric AML [40]. Recently, Ng et al. proposed a 17-gene LSC score that incorporated expressions of 17 stemness-related genes, including *KIAA0125*, and showed the scoring system was powerful to predict prognosis in AML patients [19]. Among these prognostic-relevant genes, *KIAA0125* is the only non-coding gene and expressed only in the *Homo sapiens*, but not in mice.

*KIAA0125* is located on chromosome 14 of the human genome. It was reported to be upregulated in ameloblastoma but shown as a tumor suppressor gene in colorectal cancer [59, 60]. Nonetheless, the clinical relevancy and biological role of *KIAA0125* in tumorigenesis were still largely unclear.

In this study, we found that the expression level of *KIAA0125* in BM was significantly higher in AML patients than normal HSC transplant donors. The expression of *KIAA0125* was lower in patients with t(8;21) and t(15;17) which are associated with more differentiated AML subtypes, but higher in patients with *RUNX1*, *ASXL1* mutations, *NPM1*-/*FLT3*-ITD+ or poor-risk karyotypes. It is interesting that the expression of *KIAA0125* was high in patients with *RUNX1* mutation but modest in those with *RUNX1*/*RUNX1T1* fusion consisting with the fact that AML patients with a *RUNX1* mutation usually had poor outcomes while those with *RUNX1*/*RUNX1T1* fusion had favorable prognosis. Recently, Hornung et al. identified that expression of *CD109*, *HOPX*, and *KIAA0125 genes* might be responsible for inferior survival in AML patients with *RUNX1* mutations but, on the other hand, better outcome in *RUNX1/RUNX1T1* fusion through a newly proposed statistical tool “mediation analysis.” The three genes’ expression levels were significantly higher in patients with *RUNX1* mutant but lower in those with *RUNX1*/*RUNX1T1* fusion [61]. Intriguingly, though there has been no study showing direct evidence that *RUNX1* binds to *KIAA0125* till now in the literature, *RUNX1* has been reported to bind to TGTGG core sequences as a heterodimer of *RUNX1* and CBFβ [62]. We downloaded and retrieved the DNA sequence of *KIAA0125* from the UCSC Genome Browser (https://genome.ucsc.edu/) and found several sequences of TGTGG (Supplement Table 8) within the 3000 bp upstream sequence, which might be the potential binding sites of *RUNX1*. Further studies are needed to explore the effect of the possible interaction between RUNX1 domain and *KIAA0125*.

Bioinformatics of the present study showed highly significant association of *KIAA0125* expression with stem cell signatures, either HSC or LSC. We found that expressions of *SPINK2*, *MAP7*, *HOPX*, *MMRN1*, *DNMT3B*, *TCF4*, *SLC38A1*, *DOCK1*, *ARHGAP22*, *MN1*, and 4 genes in the ATP-binding cassette (ABC) superfamily (*ABCG1*, *ABCA2*, *ABCB1*, and *ABCC1*), which have been reported to be associated with poor prognosis or chemoresistance in AML, were positively correlated to higher expression of *KIAA0125* (Fig. 3b and Table 3). *HOPX*, *DOCK1*, *DNMT3B*, *MMRN1*, and *ARHGAP22* genes were reported as important leukemia stem cell markers [19, 42, 43, 45, 50, 63]. Higher *SPINK2* expression was associated with poor prognosis in adult and pediatric AML [39, 40]. *TCF4* expression could predict outcome in *RUNX1-*mutated and translocated AML [47, 48]. *MN1* overexpression could induce AML in mice and predict ATRA resistance in human AML patients [51, 52]. Current knowledge about the association between theses *KIAA0125*-correlated genes and AML is summarized in Table 3.

Interestingly, the expression levels of several ABC transporter genes, including *ABCA2*, *ABCB1*, *ABCC1*, and *ABCG1*, were also significantly higher in AML patients with higher *KIAA0125* expression. The ABC transporter family consists of 48 proteins in subfamilies designated A to G and some of them are known to be associated with multidrug resistance via ATP-dependent drug efflux [53, 54, 57]. *ABCB1*, *ABCC1*, and *ABCG1* were reported to be responsible for chemoresistance in AML [53, 56]. The translational expression of *ABCA2* was shown to be a prognostic marker for drug resistance in pediatric acute lymphoblastic leukemia [55, 58]. The underlying mechanistic basis of the high correlation of these 4 genes to the expression of *KIAA0125* warrants further studies.

This study’s limitations lie in its retrospective nature and, crucially, the unsorted BM sample, as many cells in BM may be differentiated cells of myeloid and erythroid lineages. The study could have been more informative if we could profile *KIAA0125* expression of healthy CD34 + CD38- HSCs and more mature progenitors (CD34 + CD38- and CD34-CD117+, respectively) and compare those with leukemia blasts. Moreover, the putative oncogenic role of *KIAA0125* could be more strengthened were the expressions of *KIAA0125* investigated in AML stem cells and bulk. Despite the limitations mentioned, to the best of our knowledge, this is by far the first study specifically addressing the expression of lncRNA *KIAA0125* and its clinical and biological associations in AML patients. We found that higher *KIAA0125* expression was closely associated with *RUNX1* and *DNMT3A1* mutations in both the NTUH and TCGA cohorts. Patients with higher *KIAA0125* expression were more refractory to chemotherapy with a lower CR rate and higher refractory rate (Table 1). They had shorter OS and DFS among the total cohort and subgroups of patients with non-APL and those with normal karyotype. Based on its crucial clinical significance, further experimental studies are necessary to delineate how *KIAA0125* participates in the stem cell biology of hematopoietic lineages and its role in the pathogenesis in AML.

## Supplementary Information

ESM 1 (DOCX 680 kb)

## Data Availability

The raw and normalized microarray data reported in this article have been deposited in the Gene Expression Omnibus database (accession number GSE68469 and GSE71014).
