# Supplementary material for: Distinct clinical and biological characteristics of acute myeloid leukemia with higher expression of long noncoding RNA KIAA0125
Source: Ann Hematol. 2020 Nov 23;100(2):487–98. doi: 10.1007/s00277-020-04358-y (PMC7817567; doi:10.1007/s00277-020-04358-y)
Supplement: Supplementary file 1 — (DOCX 680 kb) [file 277_2020_4358_MOESM1_ESM.docx]

**Supplement Table 1. Expression of *KIAA0125* in various AML risk groups**

| **Risk groups** | **No.** | **Mean** | **Standard deviation** | **95% confidence interval** | |
| --- | --- | --- | --- | --- | --- |
|  |  |  |  | **Lower Bound** | **Upper Bound** |
| t(8;21) | 24 | 5.803 | 0.146 | 5.741 | 5.864 |
| t(15;17) | 27 | 6.117 | 0.757 | 5.818 | 6.417 |
| inv(16) | 9 | 7.873 | 0.993 | 7.110 | 8.637 |
| *CEBPA*^double^ | 27 | 7.164 | 0.606 | 6.924 | 7.404 |
| *NPM1*+/*FLT3*-ITD- | 57 | 7.092 | 1.025 | 6.820 | 7.364 |
| *NPM1*-/*FLT3*-ITD+ | 19 | 8.676 | 1.458 | 7.974 | 9.379 |
| *RUNX1* | 47 | 8.085 | 1.456 | 7.657 | 8.512 |
| *ASXL1* | 22 | 8.164 | 1.482 | 7.508 | 8.821 |
| Unfavorable karyotypes* | 32 | 7.685 | 1.547 | 7.127 | 8.243 |

*Based on the refined Medical research Council (MRC) classification.

**Supplement Table 2. Comparison of *KIAA0125* expression in various AML risk groups**

| Reference (I) | Comparator (J) | Mean Difference (I-J) | Std. Error | Sig. | 95% Confidence Interval | |
| --- | --- | --- | --- | --- | --- | --- |
|  |  |  |  |  | Lower Bound | Upper Bound |
| t(8;21) | t(15;17) | -.3145194 | .3276042 | .999 | -1.616661 | .987622 |
|  | inv(16) | -2.0703417^*^ | .4564382 | .010 | -3.884564 | -.256119 |
|  | *CEBPA*^doouble^ | -1.3610083^*^ | .3276042 | .031 | -2.663149 | -.058867 |
|  | *NPM1*+/*FLT3*-ITD- | -1.2887434^*^ | .2841524 | .010 | -2.418175 | -.159312 |
|  | *NPM1*-/*FLT3*-ITD+ | -2.8734680^*^ | .3585946 | .000 | -4.298788 | -1.448148 |
|  | *RUNX1* | -2.2818587^*^ | .2929723 | .000 | -3.446347 | -1.117371 |
|  | *ASXL1* | -2.3615189^*^ | .3446782 | .000 | -3.731525 | -.991513 |
|  | Unfavorable karyotypes* | -1.8820823^*^ | .3153300 | .000 | -3.135437 | -.628728 |
| t(15;17) | t(8;21) | .3145194 | .3276042 | .999 | -.987622 | 1.616661 |
|  | inv(16) | -1.7558222 | .4494693 | .059 | -3.542345 | .030701 |
|  | *CEBPA*^doouble^ | -1.0464889 | .3178228 | .217 | -2.309751 | .216773 |
|  | *NPM1*+/*FLT3*-ITD- | -.9742240 | .2728175 | .127 | -2.058602 | .110154 |
|  | *NPM1*-/*FLT3*-ITD+ | -2.5589485^*^ | .3496811 | .000 | -3.948839 | -1.169058 |
|  | *RUNX1* | -1.9673392^*^ | .2819921 | .000 | -3.088184 | -.846494 |
|  | *ASXL1* | -2.0469995^*^ | .3353951 | .000 | -3.380107 | -.713892 |
|  | Unfavorable karyotypes* | -1.5675628^*^ | .3051554 | .001 | -2.780476 | -.354650 |
| inv(16) | t(8;21) | 2.0703417^*^ | .4564382 | .010 | .256119 | 3.884564 |
|  | t(15;17) | 1.7558222 | .4494693 | .059 | -.030701 | 3.542345 |
|  | *CEBPA*^doouble^ | .7093333 | .4494693 | .961 | -1.077189 | 2.495856 |
|  | *NPM1*+/*FLT3*-ITD- | .7815982 | .4188564 | .899 | -.883246 | 2.446443 |
|  | *NPM1*-/*FLT3*-ITD+ | -.8031263 | .4725338 | .940 | -2.681324 | 1.075072 |
|  | *RUNX1* | -.2115170 | .4248892 | 1.000 | -1.900341 | 1.477307 |
|  | *ASXL1* | -.2911773 | .4620619 | 1.000 | -2.127752 | 1.545398 |
|  | Unfavorable karyotypes* | .1882594 | .4406031 | 1.000 | -1.563023 | 1.939542 |
| *CEBPA*^doouble^ | t(8;21) | 1.3610083^*^ | .3276042 | .031 | .058867 | 2.663149 |
|  | t(15;17) | 1.0464889 | .3178228 | .217 | -.216773 | 2.309751 |
|  | inv(16) | -.7093333 | .4494693 | .961 | -2.495856 | 1.077189 |
|  | *NPM1*+/*FLT3*-ITD- | .0722649 | .2728175 | 1.000 | -1.012113 | 1.156643 |
|  | *NPM1*-/*FLT3*-ITD+ | -1.5124596^*^ | .3496811 | .019 | -2.902350 | -.122569 |
|  | *RUNX1* | -.9208504 | .2819921 | .227 | -2.041695 | .199994 |
|  | *ASXL1* | -1.0005106 | .3353951 | .355 | -2.333618 | .332597 |
|  | Unfavorable karyotypes* | -.5210740 | .3051554 | .938 | -1.733987 | .691839 |
| *NPM1*+/*FLT3*-ITD- | t(8;21) | 1.2887434^*^ | .2841524 | .010 | .159312 | 2.418175 |
|  | t(15;17) | .9742240 | .2728175 | .127 | -.110154 | 2.058602 |
|  | inv(16) | -.7815982 | .4188564 | .899 | -2.446443 | .883246 |
|  | *CEBPA*^doouble^ | -.0722649 | .2728175 | 1.000 | -1.156643 | 1.012113 |
|  | *NPM1*-/*FLT3*-ITD+ | -1.5847246^*^ | .3093460 | .001 | -2.814294 | -.355155 |
|  | *RUNX1* | -.9931153^*^ | .2300818 | .020 | -1.907630 | -.078600 |
|  | *ASXL1* | -1.0727755 | .2931006 | .105 | -2.237774 | .092223 |
|  | Unfavorable karyotypes* | -.5933389 | .2579494 | .725 | -1.618620 | .431942 |
| *NPM1*-/*FLT3*-ITD+ | t(8;21) | 2.8734680^*^ | .3585946 | .000 | 1.448148 | 4.298788 |
|  | t(15;17) | 2.5589485^*^ | .3496811 | .000 | 1.169058 | 3.948839 |
|  | inv(16) | .8031263 | .4725338 | .940 | -1.075072 | 2.681324 |
|  | *CEBPA*^doouble^ | 1.5124596^*^ | .3496811 | .019 | .122569 | 2.902350 |
|  | *NPM1*+/*FLT3*-ITD- | 1.5847246^*^ | .3093460 | .001 | .355155 | 2.814294 |
|  | *RUNX1* | .5916093 | .3174667 | .900 | -.670238 | 1.853456 |
|  | *ASXL1* | .5119490 | .3657259 | .982 | -.941716 | 1.965614 |
|  | Unfavorable karyotypes* | .9913857 | .3382090 | .382 | -.352907 | 2.335678 |
| *RUNX1* | t(8;21) | 2.2818587^*^ | .2929723 | .000 | 1.117371 | 3.446347 |
|  | t(15;17) | 1.9673392^*^ | .2819921 | .000 | .846494 | 3.088184 |
|  | inv(16) | .2115170 | .4248892 | 1.000 | -1.477307 | 1.900341 |
|  | *CEBPA*^doouble^ | .9208504 | .2819921 | .227 | -.199994 | 2.041695 |
|  | *NPM1*+/*FLT3*-ITD- | .9931153^*^ | .2300818 | .020 | .078600 | 1.907630 |
|  | *NPM1*-/*FLT3*-ITD+ | -.5916093 | .3174667 | .900 | -1.853456 | .670238 |
|  | *ASXL1* | -.0796603 | .3016589 | 1.000 | -1.278676 | 1.119355 |
|  | Unfavorable karyotypes* | .3997764 | .2676341 | .972 | -.663999 | 1.463552 |
| *ASXL1* | t(8;21) | 2.3615189^*^ | .3446782 | .000 | .991513 | 3.731525 |
|  | t(15;17) | 2.0469995^*^ | .3353951 | .000 | .713892 | 3.380107 |
|  | inv(16) | .2911773 | .4620619 | 1.000 | -1.545398 | 2.127752 |
|  | *CEBPA*^doouble^ | 1.0005106 | .3353951 | .355 | -.332597 | 2.333618 |
|  | *NPM1*+/*FLT3*-ITD- | 1.0727755 | .2931006 | .105 | -.092223 | 2.237774 |
|  | *NPM1*-/*FLT3*-ITD+ | -.5119490 | .3657259 | .982 | -1.965614 | .941716 |
|  | *RUNX1* | .0796603 | .3016589 | 1.000 | -1.119355 | 1.278676 |
|  | Unfavorable karyotypes* | .4794366 | .3234167 | .974 | -.806060 | 1.764933 |
| Unfavorable karyotypes* | t(8;21) | 1.8820823^*^ | .3153300 | .000 | .628728 | 3.135437 |
|  | t(15;17) | 1.5675628^*^ | .3051554 | .001 | .354650 | 2.780476 |
|  | inv(16) | -.1882594 | .4406031 | 1.000 | -1.939542 | 1.563023 |
|  | *CEBPA*^doouble^ | .5210740 | .3051554 | .938 | -.691839 | 1.733987 |
|  | *NPM1*+/*FLT3*-ITD- | .5933389 | .2579494 | .725 | -.431942 | 1.618620 |
|  | *NPM1*-/*FLT3*-ITD+ | -.9913857 | .3382090 | .382 | -2.335678 | .352907 |
|  | *RUNX1* | -.3997764 | .2676341 | .972 | -1.463552 | .663999 |
|  | *ASXL1* | -.4794366 | .3234167 | .974 | -1.764933 | .806060 |

*Based on the refined Medical research Council (MRC) classification.

**Supplement Table 3. Comparison of cytogenetic changes between patients with lower and higher *KIAA0125* expression among 331 patients in the NTUH cohort and 135 patients in the TCGA cohort**

| Karyotype | NTUH cohort | | | | TCGA cohort | | | |
| --- | --- | --- | --- | --- | --- | --- | --- | --- |
|  | Total***** (n=331) | High (n=164) | Low (n=168) | *P* value | Total  (n=135) | High  (n=68) | Low  (n=67) | *P* value |
| Normal | 166 | 92 (56.1%) | 74 (44%) | 0.022 | 69 | 40 (59%) | 29 (43.3%) | 0.086 |
| t (8;21) | 24 | 0 (0%) | 24 (14.3%) | <0.001 | 7 | 0 (0%) | 7 (10.5%) | 0.006 |
| t (15;17) | 27 | 3 (1.8%) | 24 (14.3%) | <0.001 | 14 | 0 (0%) | 14 (21%) | <0.001 |
| Inv (16) | 9 | 6 (3.7%) | 3 (1.8%) | 0.332 | 8 | 3 (4.4%) | 5 (7.5%) | 0.490 |
| Complex† | 33 | 20 (12.2%) | 13 (7.7%) | 0.201 | 19 | 13 (19%) | 6 (9%) | 0.140 |
| Poor risk‡ | 49 | 30 (18.3%) | 19 (11.3) | 0.089 |  |  |  |  |

*Cytogenetic data at diagnosis were available in 332 patients, including 168 with lower *KIAA0125* expression and 164 with higher *KIAA0125* expression.

†≥ 3 abnormalities

‡Based on the refined Medical research Council (MRC) classification.

**Supplement Table 4. Comparison of genetic alterations between AML patients with lower and higher BM *KIAA0125* expression in the NTUH cohort**

| Genes | Total | Number of patients with the mutation (%) | | | *P* value |
| --- | --- | --- | --- | --- | --- |
|  |  | **High *KIAA0125*** | **Low *KIAA0125*** | |  |
| *FLT3-*ITD | 84 | 50 (59.5) | | 34 (40.5) | 0.048 |
| *FLT3-*TKD | 32 | 13 (40.6) | | 19 (59.4) | 0.258 |
| *N-RAS* | 59 | 31 (52.5) | | 28 (47.5) | 0.686 |
| *K-RAS* | 15 | 6 (40) | | 9 (60) | 0.422 |
| *PTPN11* | 22 | 13 (59.1) | | 9 (40.9) | 0.386 |
| *KIT* | 15 | 6 (40) | | 9 (60) | 0.422 |
| *WT1* | 26 | 17 (65.4) | | 9 (34.6) | 0.106 |
| *NPM1* | 99 | 53 (53.5) | | 46 (46.5) | 0.425 |
| *CEBPA*^double^ | 27 | 13 (48.1) | | 14 (51.9) | 0.829 |
| *RUNX1* | 50 | 32 (64) | | 18 (36) | 0.034 |
| *MLL/*PTD | 13 | 8 (61.5) | | 5 (38.5) | 0.429 |
| *ASXL1* | 52 | 26 (50) | | 26 (50) | 0.982 |
| *IDH1* | 20 | 14 (70) | | 6 (30) | 0.067 |
| *IDH2* | 51 | 26 (51.0) | | 25 (49.0) | 0.897 |
| *TET2* | 56 | 25 (44.6) | | 31 (55.4) | 0.369 |
| *DNMT3A* | 66 | 42 (63.6) | | 24 (36.4) | 0.015 |
| *TP53* | 16 | 8 (50) | | 8 (50) | >0.999 |

**Supplement Table 5. Comparison of genetic alterations between AML patients with lower and higher BM *KIAA0125* expression in the TCGA cohort**

| Genes | Total | Number of patients with the mutation (%) | | | *P* value |
| --- | --- | --- | --- | --- | --- |
|  |  | **High *KIAA0125*** | **Low *KIAA0125*** | |  |
| *DNMT3A* | 26 | 21 (80.8) | | 5 (19.2) | <0.001 |
| *RUNX1* | 13 | 11 (84.6) | | 2 (15.4) | 0.017 |
| *PTPN11* | 5 | 5 (100) | | 0 (0) | 0.058 |
| *TP53* | 10 | 8 (80) | | 2 (20) | 0.098 |
| *PCLO* | 5 | 1 (20) | | 4 (80) | 0.209 |
| *ASXL1* | 3 | 3 (100) | | 0 (0) | 0.245 |
| *KIT* | 9 | 3 (33.3) | | 6 (66.7) | 0.327 |
| *BCORL1* | 5 | 4 (80) | | 1 (20) | 0.367 |
| *CMYA5* | 4 | 1 (25) | | 3 (75) | 0.367 |
| *FLT3-*ITD/TKD | 16 | 10 (62.5) | | 6 (37.5) | 0.429 |
| *NRAS* | 6 | 2 (33.3) | | 4 (66.7) | 0.442 |
| *KRAS* | 6 | 2 (33.3) | | 4 (66.7) | 0.442 |
| *IDH1* | 9 | 6 (66.7) | | 3 (33.3) | 0.494 |
| *WT1* | 11 | 7 (63.6) | | 4 (36.4) | 0.533 |
| *MUC16* | 12 | 5 (41.7) | | 7 (58.3) | 0.564 |
| *SMC3* | 8 | 5 (62.5) | | 3 (37.5) | 0.719 |
| *IDH2* | 10 | 6 (60) | | 4 (40) | 0.745 |
| *NPM1* | 16 | 9 (56.3) | | 7 (43.7) | 0.792 |
| *NF1* | 6 | 3 (50) | | 3 (50) | >0.999 |
| *TET2* | 5 | 3 (60) | | 2 (40) | >0.999 |

**Supplement Table 6. Univariate analysis (Cox regression) of the impact of different variables on the overall survival in the 227 AML patients who received standard treatment**

| **Variable** | **HR** | **Lower 95% CI** | **Upper 95% CI** | ***P* value** |
| --- | --- | --- | --- | --- |
| **WBC*** | 1.000 | 1.000 | 1.000 | 0.394 |
| **Karyotype**† | 1.443 | 1.111 | 1.874 | 0.006 |
| ***FLT3-*ITD** | 2.134 | 1.342 | 3.394 | 0.001 |
| ***FLT3-*TKD** | 1.324 | 0.680 | 2.578 | 0.409 |
| ***N-RAS*** | 0.908 | 0.478 | 1.723 | 0.767 |
| ***K-RAS*** | 1.770 | 0.646 | 4.855 | 0.267 |
| ***PTPN11*** | 1.910 | 0.828 | 4.405 | 0.129 |
| ***KIT*** | 0.914 | 0.333 | 2.510 | 0.862 |
| ***WT1*** | 1.902 | 0.939 | 3.850 | 0.074 |
| ***NPM1*** | 1.262 | 0.776 | 2.051 | 0.348 |
| ***CEBPA*^double^** | 0.312 | 0.098 | 0.992 | 0.048 |
| ***RUNX1*** | 2.292 | 1.300 | 4.041 | 0.004 |
| ***MLL/*PTD** | 3.769 | 1.490 | 9.533 | 0.005 |
| ***ASXL1*** | 1.289 | 0.592 | 2.808 | 0.523 |
| ***IDH1*** | 1.385 | 0.601 | 3.190 | 0.445 |
| ***IDH2*** | 0.771 | 0.354 | 1.679 | 0.512 |
| ***TET2*** | 1.428 | 0.785 | 2.598 | 0.243 |
| ***DNMT3A*** | 1.599 | 0.918 | 2.785 | 0.098 |
| ***TP53*** | 6.628 | 2.378 | 18.469 | <0.001 |
| ***KIAA0125*** | 2.181 | 1.362 | 3.493 | 0.001 |

*P* values < .05 are considered statistically significant.

Abbreviations: HR, hazard ratios; CI, confidence interval.

*As continuous variable.

†Unfavorable cytogenetics versus others. The classification of favorable, intermediate and unfavorable cytogenetics is based on the refined Medical Research Council (MRC) classification. Favorable: t(15;17)(q22;q21), t(8;21)(q22;q22), and inv(16)(p13q22)/t(16;16)(p13;q22); unfavorable: abn(3q) (excluding t(3;5)(q25;q34)), inv(3)(q21q26)/t(3;3)(q21;q26), add(5q)/del(5q), -5, -7, add(7q)/del(7q), t(6;11)(q27;q23), t(10;11)(p1113;q23), other t(11q23) (excluding t(9;11)(p21~22;q23) and t(11;19)(q23;p13)), t(9;22)(q34;q11), -17, and abn(17p); and intermediate: entities not classified as favorable or adverse. Seven patients without chromosome data were not included in the analysis.

**Supplement Table 7. Multivariable analysis for DFS and OS in 227 AML patients who received standard treatment**

|  | DFS | | | | OS | | | |
| --- | --- | --- | --- | --- | --- | --- | --- | --- |
|  | **95% CI** | | | | **95% CI** | | | |
| Variable | **HR** | **Lower** | **Upper** | ***P*** | **HR** | **Lower** | **Upper** | ***P*** |
| Age* | 1.008 | 0.996 | 1.019 | 0.198 | 1.031 | 1.014 | 1.047 | <0.001 |
| WBC* | 1.004 | 1.001 | 1.007 | 0.002 | 1.004 | 1.001 | 1.008 | 0.016 |
| Karyotype† | 1.711 | 1.270 | 2.304 | <0.001 | 1.868 | 1.258 | 2.773 | 0.002 |
| *NPM1/FLT3-*ITD‡ | 0.580 | 0.320 | 1.050 | 0.072 | 0.878 | 0.436 | 1.770 | 0.716 |
| *CEBPA*^double^ | 0.531 | 0.273 | 1.035 | 0.063 | 0.314 | 0.096 | 1.028 | 0.056 |
| *RUNX1* | 1.409 | 0.805 | 2.468 | 0.230 | 1.351 | 0.689 | 2.648 | 0.381 |
| *MLL*-PTD | 2.189 | 0.997 | 4.803 | 0.051 | 2.597 | 0.960 | 7.030 | 0.060 |
| *TP53* | 1.955 | 0.708 | 5.399 | 0.196 | 3.048 | 0.959 | 9.692 | 0.059 |
| Higher *KIAA0125* expression* | 1.345 | 1.188 | 1.524 | <0.001 | 1.332 | 1.127 | 1.573 | 0.001 |

*P* values < .05 are considered statistically significant.

Abbreviations: HR, hazard ratios; CI, confidence interval.

*As continuous variable.

†Unfavorable cytogenetics versus others. The classification of favorable, intermediate and unfavorable cytogenetics is based on the refined Medical Research Council (MRC) classification. Favorable: t(15;17)(q22;q21), t(8;21)(q22;q22), and inv(16)(p13q22)/t(16;16)(p13;q22); unfavorable: abn(3q) (excluding t(3;5)(q25;q34)), inv(3)(q21q26)/t(3;3)(q21;q26), add(5q)/del(5q), -5, -7, add(7q)/del(7q), t(6;11)(q27;q23), t(10;11)(p1113;q23), other t(11q23) (excluding t(9;11)(p21~22;q23) and t(11;19)(q23;p13)), t(9;22)(q34;q11), -17, and abn(17p); and intermediate: entities not classified as favorable or adverse. Seven patients without chromosome data were not included in the analysis.

‡*NPM1*+/*FLT3*-ITD- versus other subtypes.

**Supplement Table 8. The 3000bp upstream sequence of *KIAA0125*.**

Several occurrences of TGTGG motif sequence (highlighted in yellow), which might be the potential binding sites of *RUNX1*, were found in the 3000bp upstream sequence of KIAA0125.

| AGTCAGGTGAGGCCGAGCTCAGGTGAAGCCCAGAGGTGAGGTCTAGGCCA  GGTGAGGTCCAGGCCAGGTGAGGTCCAGGTCAGGTGAGGCCCAGGTCAGG  CAAGGCTGAGGTAGATGTATGAGACTTCTGTAATTTTCAGTTGGTGCCAA  CCCTGCCTGGTGTCCCTGCCCCTCCTCCCAGCCCATGCTCTGTGCCTGCC  AGATGGCGGCCCCTGCACAGGTGCTGCTGGCTGTGGAGGAGCTGGGCTCT  GCCTCCCTGTGCATGGGCGTCCCTCTCGGGCTCTGGCCTGGGAGTGTGGC  TGAGTTGCTTGTCTCCGGAATGTACCAACTGTGCCGTCCTTGGGGGTATA  TGTCCTCGGGGGGATACGGCTCTGTGCCTGCTCCACATCAGGCCCCAGGA  GCTGCCAGCAGGTACCAGCCTGCCCTGCCACACAGTGTGCCTGCAGCCTG  TCCGGGGATGCCCAGGGAGGTGAGTGCCACCACATATCAGGCCTTTTCTC  TTTAAAGTCATTTCTTTGGGGATACATCATCAATGTCTCATATACTGAAT  GTATGTCTGTATCATTGTGCAATTGCCTGTGTCATCGTTTATTTATCCAA  CCTGGGTTAATGTCTTTGCTATTATGAACAGTGCTGGACTGAGAATTTTC  TAAACACAGCTGTGTGCATTTTCCTCTTCTTGCAATTTAGAATTTTAACT  GCTGTTTTCAAGGTACTGTAATGTATTTGTTCTCTTCTTGTTAGGAGACT  TGCCAACCCTGTGTGTCTCAGTTCATACCCTCTTCCTTCCCCAGTAGAAG  TAACGACCACTGTGTTTATGTGATCATCCTTTTCTTGATTTTCCTTATAG  TTTTCCTAGTGGAAAGTTTATCCCTTAAGAAGATAGTTCATTTTGCCGGC  TGTAAATTTTATTTAGAAGAAATCACATTGAAAGTATTTTTTGGACTTTC  CTTTGTTACTCCAATTACTCAGCATTGTCATGAACTCAACCACAGAGTCA  CCTGTAACCCTCACTGTTGTCCTTCTGGCTGTCTGGGTTTGCATTTCATG  AACCTGCCATCGTTTATTTGCCTGTTTTCCTTCAGATGGCTGTTTGCTTC  ATTCTCAGTTTGGGGCTATGACAAACATATGTTCTGCACATCTTTGCCCA  TGAGGCTCTCAGGGAGGGCTCTGGAGCTGGCATTGCCTGCAGGGCTCTGC  TTTGTTGCAGGGAGTTCCTGCCAAGGCTTTTCAGAGTGTCTGTGCCCAGC  CTGAAGGTACACACTGTACTTTGCCCTTGCATCAGGCACTTTCCTTGTGC  TTGCTTCTGTGTGGCTCCACATTCTGGAGAATTTATTCAGATCTGTGCTG  CAAATCCATTTCACTGATTCTCTCTTTAGCTGTGTCTACATCAGCTGTTA  AGCATCCCATGATGCAGCATTGTGGGCACAGGGCAAACTCTTGAAAGATG  ACAGTGTAGGATAGCGGCTGCTTCTCCTTCCCTGTGCCCTTCCCACACTG  TCCTCCTGGGCTCACTCCCAGCCATCGATCTTGAACACCAGTTTATGGAA  CTATCTGCACAGGAAAGCAGAAACAGCAAAAGGCCCTGCTCAGGCTCTGC  CCGCATCCCCTCTTGCACACCCGCCAAAGCTCTTTCCTTGGGGCCTGTGC  AAGCTTCCCAGCTGCTTCTCATTTTCTGTTTACTCTGCTCACTGGCCAGT  GGGGTGATGTCTGGGGGGGAGTCTGGTGCGTTTTGGGCATTGATGGCCAC  CCCTAGGCCCTACTTCCCAGACGCTCCCCCCAGCCCCTCAGCTCCAGAAG  TGGAAGCGTTTACAGCAGGGCTTTGGGAATGGGGCTGTGTCACTGTGGGC  ATAGCAGCTGGTACTACTACAATATCCTCACAGTGACACGAGCCCCCACA  AAATCCTCCTGTCCCCGCGGGAGTCACTGAGTCCCCTCTTGCTGTCTCTG  GCTAGTTCTCCTGCTGATACTATGATTTCCAGGGGGTTTTTGTCTGAAAC  TCAGGGTGTGTTGGAGAGGACTCTGAGCCCAGTGCTGTACAGGGGGCTCC  TCCTTTGTCCTGGGGGAGTTGCGTGGACCCTGTTTTTGGTCAAGGGAAGT  ACTTGCTGGTGAAGGAGACCTCCCCTCCTTTCTTTCTCAGGAGCCCCCTC  TGATGCCGTTGCCTGGTGTTTCTCAGGGCTGGTGCTGGGGGCTCAGCAGT  GTCTGCCCTGTTCCAGGTGGGAATGTGGGTCTGTTCTGTTTCCACGCGGT  GTTCTGGGGCCGCCAGTGAGGGGCTCGGGATGTCAGCGGCTGGTCTCTGT  CCCTATGGTCTGGGCTCCGGTTCACTGCTCCCCTGCCCTCCAGGTCGGTC  ACTGACTCAGTTACTATCCAGCGGGCTCCGTGGCTGTTCAGTGGTGGCTG  CAGGTCTCTTCCCAGGAGAGGCCTGCGAGAGGGCTGGGCTGTCTGGGAGC  CCTGCATTCTCCCATGATGTTGCTGCCTGGATCCCTCGTCTTTACAGGGA  GTGCCGAGCCTCCCTGCAGGTGCGGGCAGTGAGAGACACAGGCGGACGTG  CATCAGGGTGCTGGAGGCCGATTTCTTTCAGTGCCTTCTGCCTGTGGAAG  GGCTGAGCTCCCTGCTTCTGTGCACAGGAGGCTGCCGTGTAACCGGGCAG  TGAGGGCAAGGGCCTGCATGGGGAAGACTTGGGTGAGCCTTTGTCCTGGA  AATACCAGGGCTGGGTCCAAGAGGGGAGCAGGGTCAGAGTGTCCAGGAGG  AAGGTGAGGGCATGGGCAGTGTGGGGGTGGGAGTGCACGGTCAGTGCCAT  GGCTCAGGGGCCCCAGGAGAGGAAGAGCTCGAGTTGTGGGCAGGAGGAGG  CAGGGGGTAGGCACAGAGGGTAGAAACTGAGGCTCTGGCAGCAGAAAAGG  GGAGGGCCTGCATGTGCAGGGTTGGCCTGGGAGGGGTGTCTGGAGGGAGA  GACAGGGGTCTGGGTGGAGACCAGGGTGTAGGCTGCAGGGATGGGACCCC |
| --- |

**Supplement Figure 1. Dot plots depicting distribution of *KIAA0125* expression in 347 AML patients.**

**
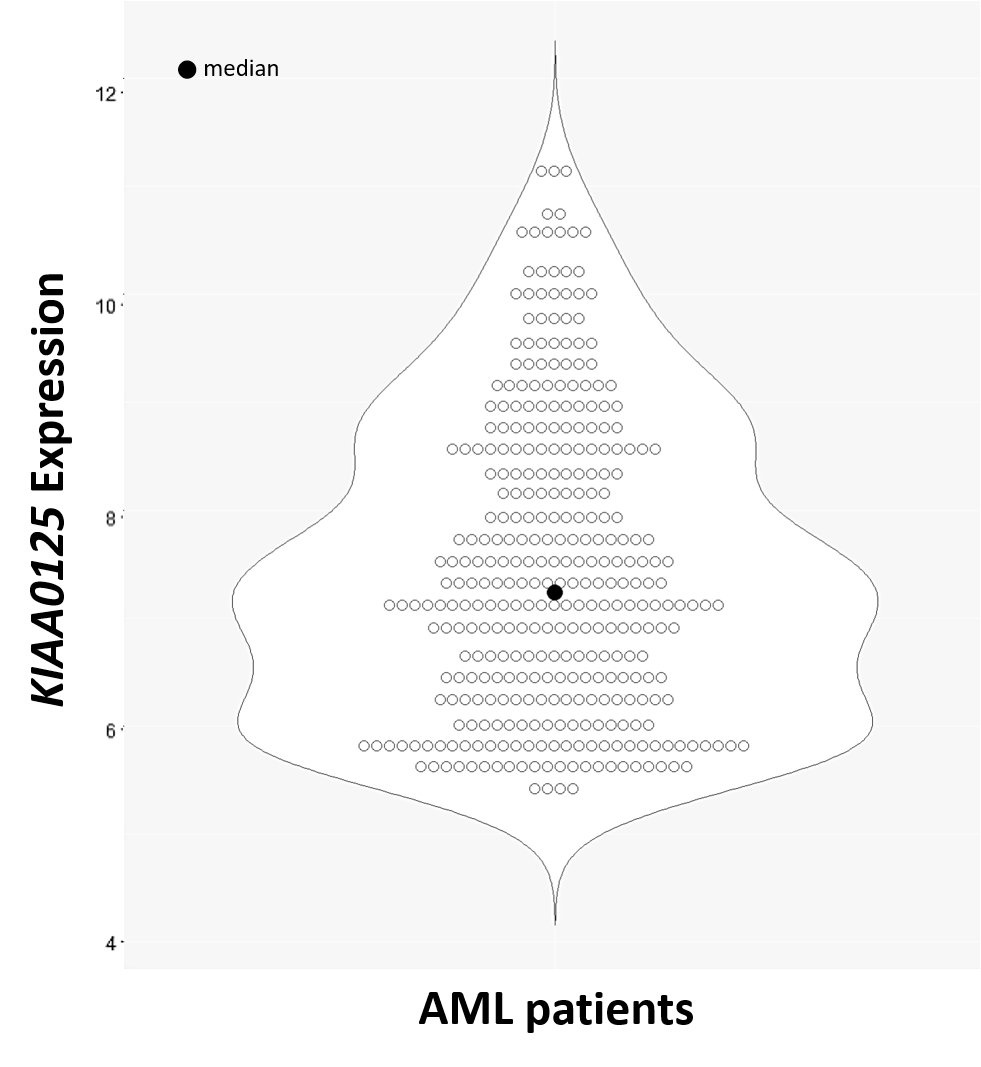
**

**Supplement Figure 2. Box plots depicting expression of *KIAA0125* in AML patients with different genotypes.**

Among 166 cytogenetically normal AML patients, those with *FLT3*-ITD (a) or *RUNX1* mutation (b) had higher *KIAA0125* expression in the bone marrow than others. Patients with *DNMT3A* mutation (c) had a trend of higher *KIAA0125* expression than those without the mutation.

| **a** | **b** |
| --- | --- |
| 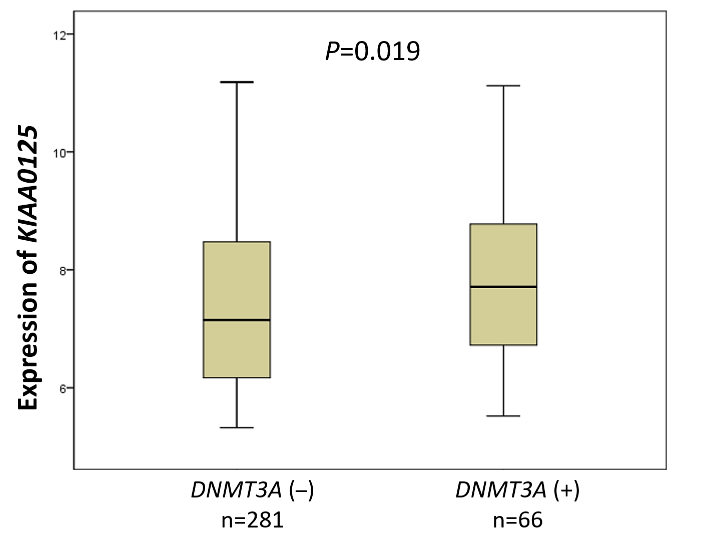 | 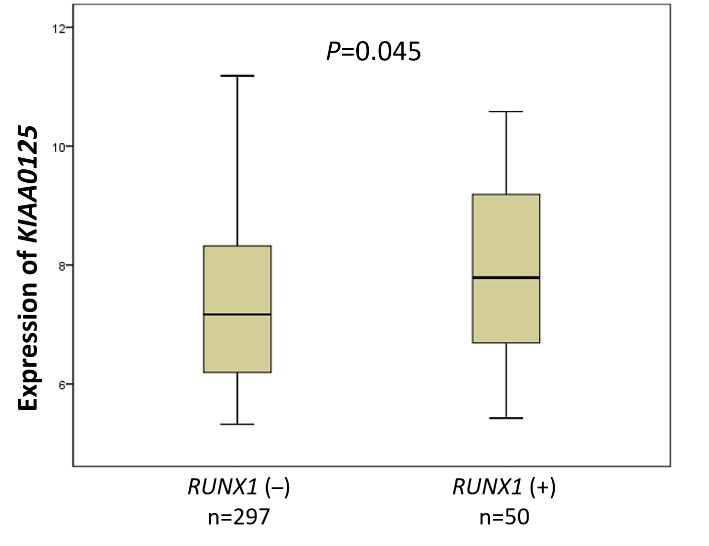 |
| **c** |  |
| 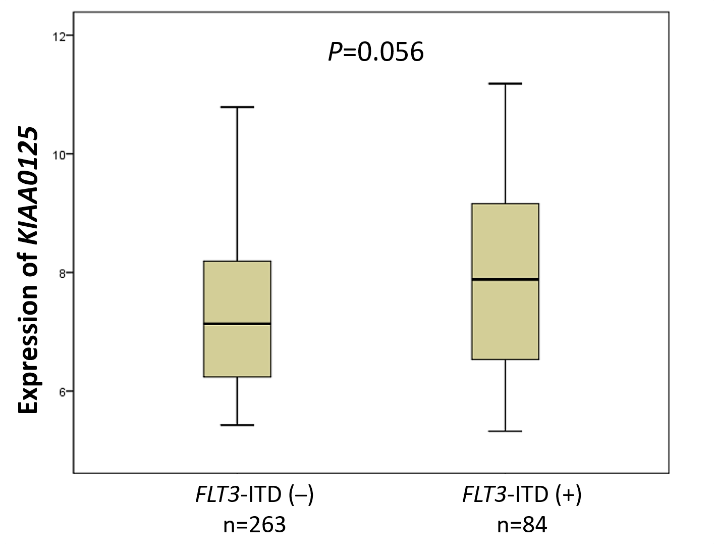 |  |

**Supplement Figure 3. Distribution and maximally selected rank statistics of *KIAA0125* expression in the NTUH cohort, TCGA, and GSE12417.**

| **NTUH** | **TCGA** | **GSE12417** |
| --- | --- | --- |
| Optimal cut point: 7.72 | Optimal cut point: 8.56 | Optimal cut point: 9.71 |
| **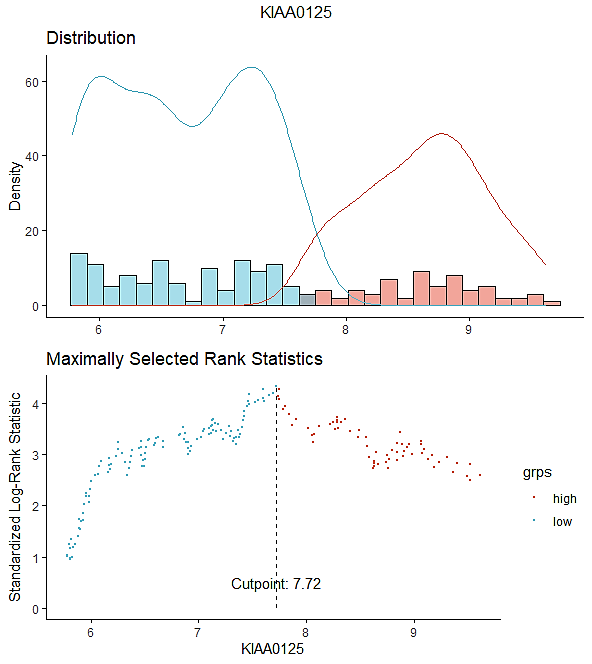** | **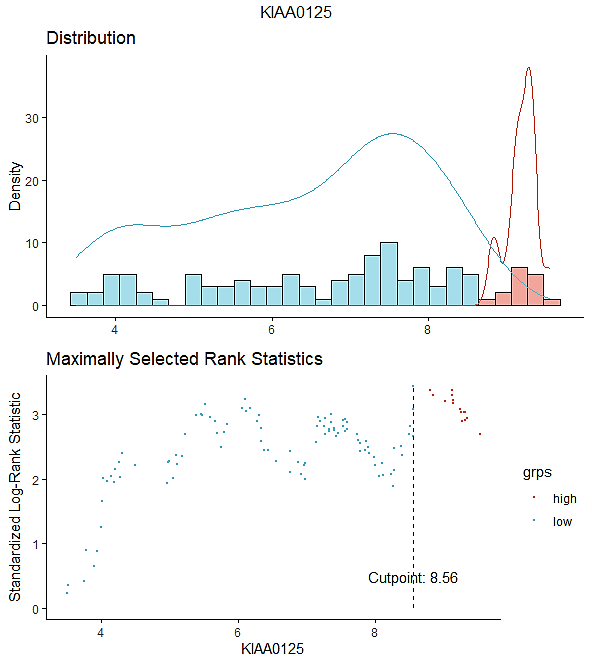** | **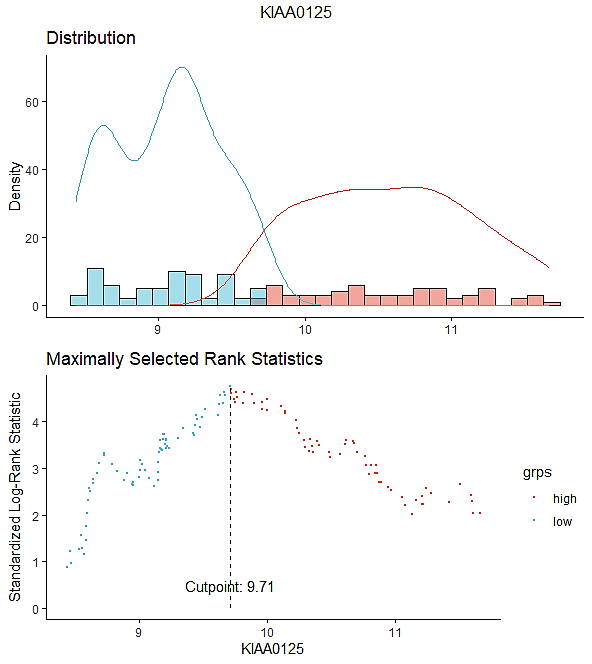** |

**Supplement Figure 4. Kaplan-Meier plots stratified by expression of *KIAA0125*.**

DFS (a) and OS (b), not censored at HSCT, of the 227 AML patients receiving standard induction chemotherapy in NTUH cohort. Patients with higher *KIAA0125* expression had worse clinical outcomes than those with lower expression.

| **a** | | **b** | |
| --- | --- | --- | --- |
| **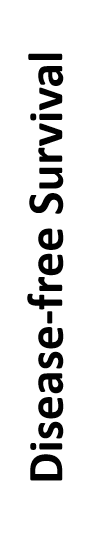** | **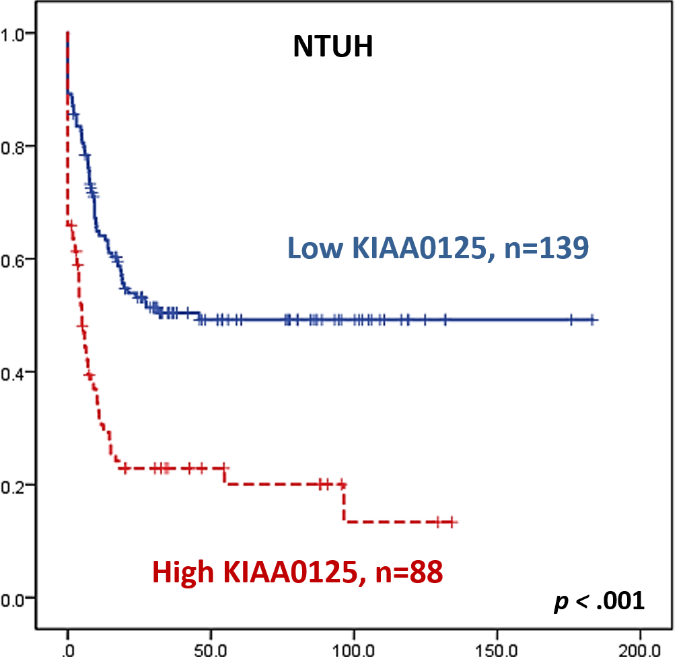** | **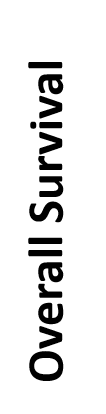** | **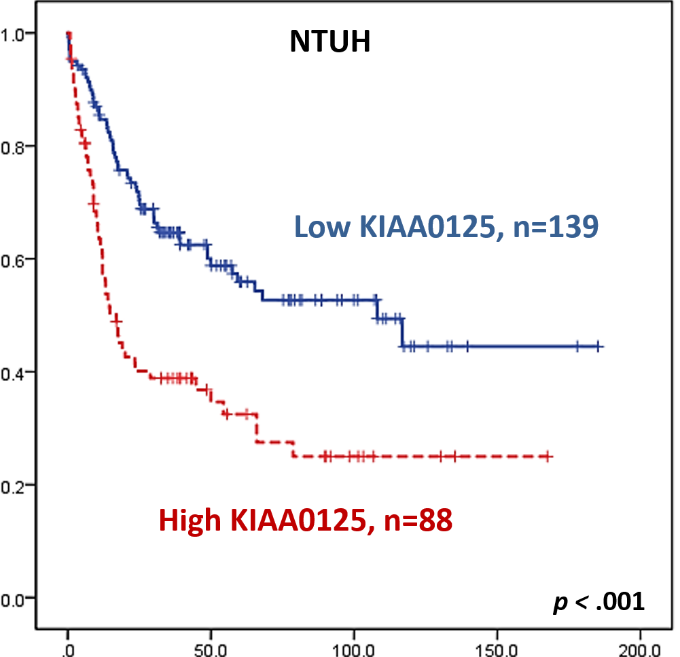** |
|  | **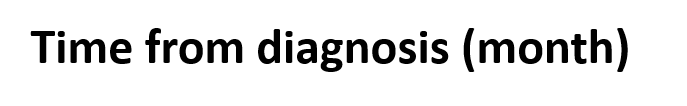** |  | **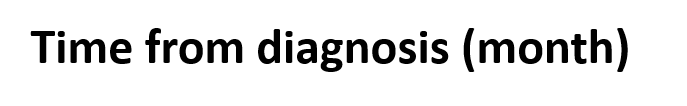** |
